# Congenitally corrected transposition

**DOI:** 10.1186/1750-1172-6-22

**Published:** 2011-05-14

**Authors:** Gonzalo A Wallis, Diane Debich-Spicer, Robert H Anderson

**Affiliations:** 1 Congenital Heart Center at the University of Florida, Gainesville, Florida, USA; 2 University of Florida, Gainesville, Florida, USA; 3 The Congenital Heart Institute of Florida (CHIF), St. Petersburg, Florida, USA; 4 University College, London. UK

## Abstract

Congenitally corrected transposition is a rare cardiac malformation characterized by the combination of discordant atrioventricular and ventriculo-arterial connections, usually accompanied by other cardiovascular malformations. Incidence has been reported to be around 1/33,000 live births, accounting for approximately 0.05% of congenital heart malformations. Associated malformations may include interventricular communications, obstructions of the outlet from the morphologically left ventricle, and anomalies of the tricuspid valve. The clinical picture and age of onset depend on the associated malformations, with bradycardia, a single loud second heart sound and a heart murmur being the most common manifestations. In the rare cases where there are no associated malformations, congenitally corrected transposition can lead to progressive atrioventricular valvar regurgitation and failure of the systemic ventricle. The diagnosis can also be made late in life when the patient presents with complete heart block or cardiac failure. The etiology of congenitally corrected transposition is currently unknown, and with an increase in incidence among families with previous cases of congenitally corrected transposition reported. Diagnosis can be made by fetal echocardiography, but is more commonly made postnatally with a combination of clinical signs and echocardiography. The anatomical delineation can be further assessed by magnetic resonance imaging and catheterization. The differential diagnosis is centred on the assessing if the patient is presenting with isolated malformations, or as part of a spectrum. Surgical management consists of repair of the associated malformations, or redirection of the systemic and pulmonary venous return associated with an arterial switch procedure, the so-called double switch approach. Prognosis is defined by the associated malformations, and on the timing and approach to palliative surgical care.

## Disease names, Synonyms, Abbreviations and Acronyms

Congenitally corrected transposition of the great arteries, corrected transposition of the great arteries, L-transposition of the great arteries, ventricular inversion.

## Definition

**Figure 1:**
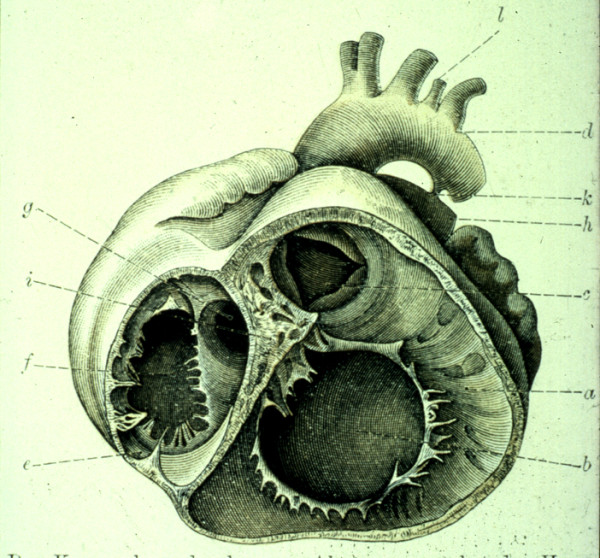
**This illustration from the atlas of the Baron von Rokitansky shows the short axis of the ventricular mass viewed from the ventricular aspect in a specimen with congenitally corrected transposition**.

The Baron Rokitansky, in his atlas of 1875, was the first to describe the entity we now know as congenitally corrected transposition (Figure 1). It is also known as "L-transposition" or ventricular inversion, although, as we will describe, these terms are less than precise.

**Figure 2:**
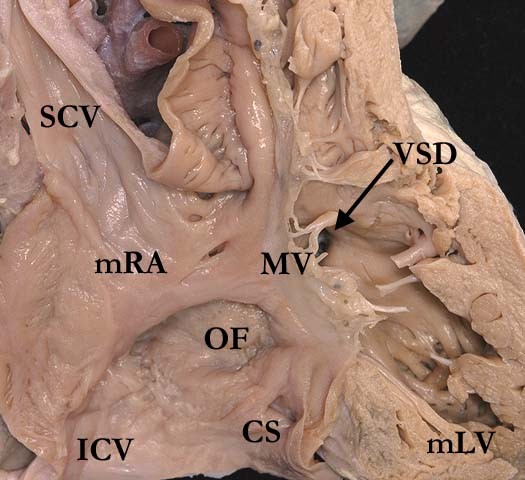
**The superior caval vein (SCV) and inferior caval vein (ICV) are connected to the morphologically right atrium (mRA), which in turn empties through the mitral valve (MV) to the morphologically left ventricle (mLV) and thence to the pulmonary trunk**. There is a ventricular septal defect (VSD) present below the pulmonary valve. The right atrium shows the normal position of the oval fossa (OF) and the coronary sinus (CS).

**Figure 3:**
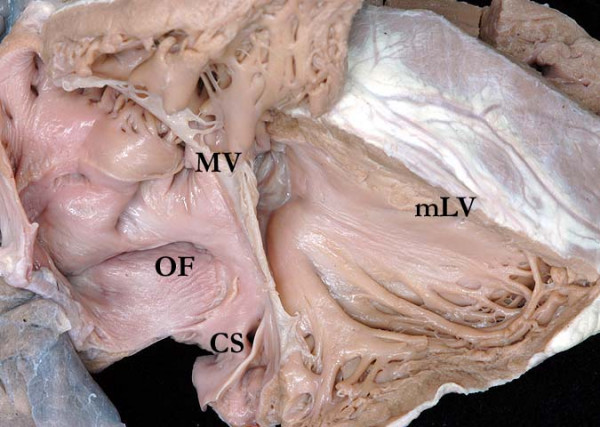
**This dissection is made to replicate the parasternal long echocardiographic projection**. It shows the coronary sinus (CS) opening to the right-sided morphologically right atrium, which connects to the morphologically left ventricle (mLV) through the mitral valve (MV). The oval fossa (OF) is clearly seen in the atrial septum

**Figure 4:**
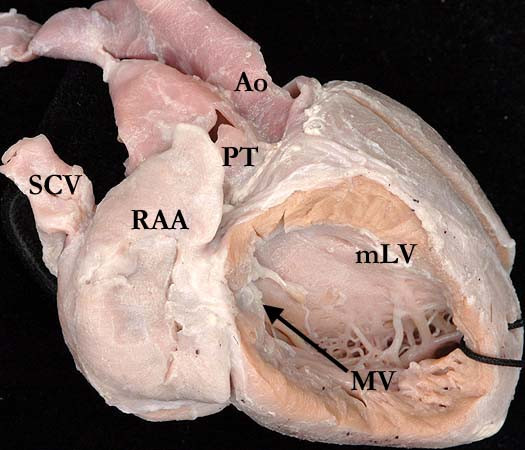
**This illustration demonstrates how the morphologically right atrium, with its characteristic appendage (RAA), is connected to a morphologically left ventricle (mLV) across the mitral valve (MV), with the ventricle then connected to the pulmonary trunk (PT)**. Note the smooth septal surface of the ventricle.

**Figure 5:**
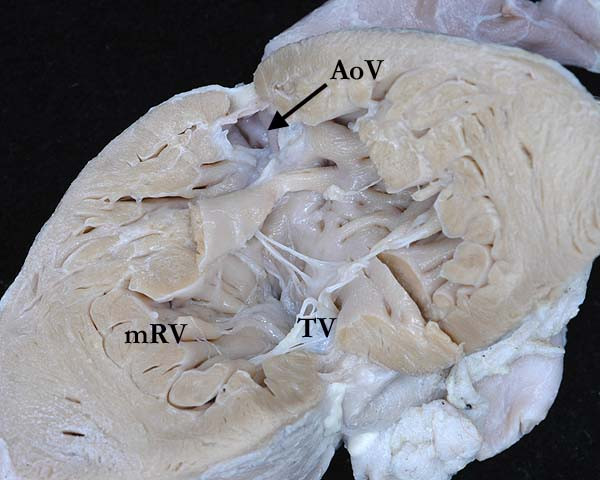
**This illustration shows the morphologically left atrium connected to the morphologically right ventricle (mRV) across the tricuspid valve (TV), with the ventricle giving rise to the aortic valve**. The ventricle possesses coarse trabeculations, with the leaflets of the tricuspid valve attached directly to the muscular ventricular septum.

The essence of the lesion is the combination of discordant atrioventricular and ventriculo-arterial connections (Figure 2 and Figure 3). Thus, the morphologically right atrium is connected to a morphologically left ventricle across the mitral valve, with the left ventricle then connected to the pulmonary trunk (Figure 4). The morphologically left atrium is connected to the morphologically right ventricle across the tricuspid valve, with the morphologically right ventricle connected to the aorta (Figure 5). When the atrial chambers are arranged in their usual fashion, the morphologically left ventricle is usually positioned to the right, and the aorta, arising from the right ventricle, is left-sided. The malformed heart can also be found with the atrial chambers in mirror-image arrangement. In this setting, the morphologically left ventricle is left-sided, and the aorta is typically positioned to the right. Hence, such patients do not have "L-transposition", but do have congenitally corrected transposition.

Congenitally corrected transposition is also described as "double discordance". As the name implies, the discordant connections at both the atrioventricular and ventriculo-arterial junctions results in normal physiology - hence the congenital correction of the transposition. Because of the double discordance, the systemic venous return is pumped to the lungs, while the pulmonary venous return is directed to the body. Symptoms are produced not by the segmental arrangement of the cardiac components, but by the presence of associated anomalies. A triad of malformations, made up of an interventricular communication, obstruction of the outlet from the morphologically left ventricle, and anomalies of the morphologically tricuspid valve, are sufficiently constant to be considered as part of the malformation. Abnormalities of atrioventricular conduction are also frequent, but any other malformation that might co-exist should be anticipated to be present at some time or in some place.

## Epidemiology

Congenitally corrected transposition is a rare cardiac condition, with the incidence reported to be 1/33,000 live births, thus accounting for approximately 0.05% of congenital cardiac malformations [1-3].

## Aetiology

The aetiology of congenitally corrected transposition is not currently known. Based on geographic incidence, studies have suggested an association with environmental factors, such as hair dye and air pollutants [4]. Recently studies have shown an increase in incidence among families with previous cases of congenitally corrected transposition, with a recurrence risk in siblings of between 2.6% to 5.2%.

## Anatomy and Morphogenesis

**Figure 6:**
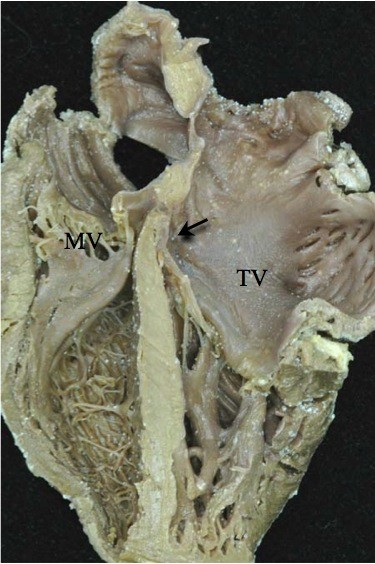
**Note the reversed off-setting of the attachments of the leaflets of the atrioventricular valves to the septum (Arrow), with the mitral valve (MV) on the right side attached appreciably higher that the tricuspid valve (TV) on the left side**.

**Figure 7:**
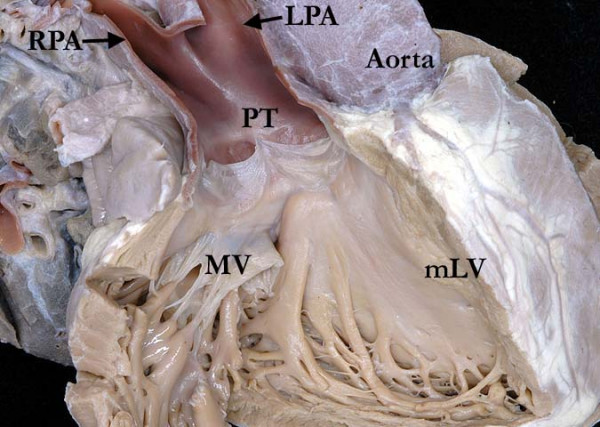
**The image shows the fibrous continuity between the leaflets of the pulmonary trunk (PT) and mitral valves (MV) in the roof of the right-sided morphologically left ventricle (mLV)**.

**Figure 8:**
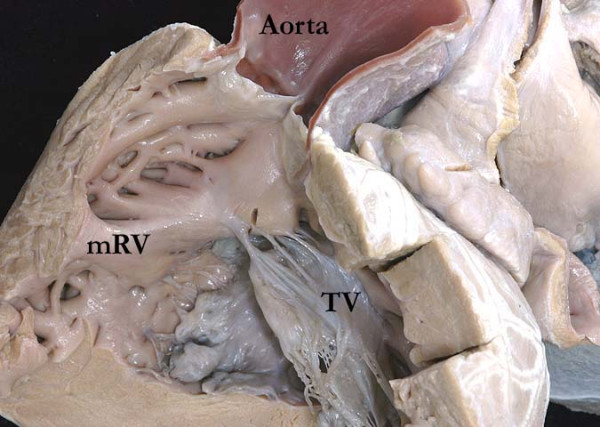
Note how the aorta is supported by a complete muscular infundibulum above the coarsely trabeculated morphologically right ventricle (mRV), the infundibulum interposing between the hinges of the aortic and tricuspid (TV) valves.

When the atrial chambers are in their expected positions, the ventricular mass is mirror-imaged, this arrangement known as left hand ventricular topology, or left handed ventricular looping. With this arrangement, when the ventricular septum is intact, there is reversed off-setting of the attachments of the leaflets of the atrioventricular valves to the septum, with the mitral valve on the right side attached appreciably higher that the tricuspid valve on the left side at the crux of the heart (Figure 6). Almost always there is fibrous continuity between the leaflets of the pulmonary and mitral valves in the roof of the right-sided morphologically left ventricle (Figure 7). The pulmonary valve is wedged between the atrial septum and the mitral valve, deviating the atrial septum away from the ventricular septum, and producing an abnormal arrangement of the atrioventricular conduction axis. The morphologically right ventricle, receiving the pulmonary venous return, pumps into the aorta, which is supported by a complete muscular infundibulum (Figure 8), with the aortic valve located anterior and to the left of the pulmonary trunk (Figure 4 and 7). In a small number of patients with usual atrial arrangement, the aorta can be found in right-sided position, or directly anterior to the pulmonary trunk, again pointing to the inappropriateness of using "L-transposition" as a name for the entity.

Paired papillary muscles, located in infero-medial and supero-lateral position, typically support the leaflets of the mitral valve. The supero-lateral papillary muscle is potentially vulnerable during a surgical ventriculotomy, so the surgeon will often approach the ventricle either through an atrioventricular or an arterial valve. In the minority of hearts with an intact ventricular septum, the membranous septum can be extensive. Because of the discordant connections, its interventricular component separates the morphologically left atrium from the morphologically left ventricle. There is a characteristic prominent recess seen antero-superiorly within the morphologically left ventricle. This feature was useful in recognizing the abnormality in the days when the diagnosis was made angiographically.

The ventricular mass with usual arrangement of the great vessels, showing left hand topology, also differs from the usual pattern because the outflow tracts arise parallel to one other by virtue of the discordant ventriculo-arterial connections, rather than crossing as they do with concordant ventriculo-arterial connections. The ventricles are positioned side by side, often with an added supero-inferior obliquity. Excessive tilting produces a supero-inferior relation of the ventricles [5]. The ventricular mass has an abnormal orientation in relation to the thorax, with the apex frequently pointing to the right in the setting of usual atrial arrangement.

The coronary arteries arise from the two aortic sinuses that are adjacent to the pulmonary trunk, their precise position varying in relation to the location of the aortic root. It is not uncommon to find all three coronary arteries arising from one aortic sinus. The epicardial distribution of the coronaries is constant, following their respective ventricles. Thus, in the setting of usual atrial arrangement the right-sided coronary artery will exhibit the pattern of a morphologically left coronary artery, with its short main stem dividing into anterior interventricular and circumflex branches. The circumflex artery will encircle the mitral valvar orifice. The left-sided coronary artery will be arranged as a morphologically right artery, giving origin to the infundibular and marginal branches as it encircles the tricuspid valve. The anterior interventricular artery is an excellent guide to the location of the muscular ventricular septum.

The atrioventricular conduction system is abnormally positioned due to the malalignment of the atrial and ventricular septal structures, the gap being filled by an extensive membranous septum when the septal structures are intact. Due to this relationship, it is impossible for the penetrating atrioventricular bundle to take its origin from the regular atrioventricular node, located at the apex of the triangle of Koch in the base of the atrial septum. Instead, the atrioventricular node is located beneath the opening of the right atrial appendage, positioned at the lateral margin of the area of pulmonary-to-mitral valvar fibrous continuity. This places the conduction axis in direct relationship to the leaflets of the pulmonary valve. The long non-branching bundle descends down the anterior surface of the subpulmonary outflow tract, and bifurcates into a cord-like right bundle branch, which extends leftward to reach the morphologically right ventricle. The fan-like left bundle branch cascades down the smooth surface of the morphologically left ventricle. This abnormal course of the conduction system is of crucial significance to the surgeon in the presence of a ventricular septal defect or subpulmonary obstruction, since the electrical system of the heart is vulnerable during any attempted surgical repair of the ventricular septal defect, with the potential for producing traumatic heart block.

## Associated Malformations

The majority of cases of congenitally corrected transposition have one or more coexisting malformations. The most common are an interventricular communication, seen in up to three-fifths of patients, obstruction of the pulmonary outflow tract, found in two-fifths of patients, and abnormalities of the morphologically tricuspid valve, seen in up to nine-tenths of patients [6].

### The Ventricular Septal Defect

The majority of the defects are perimembranous, located below the pulmonary valve, with the diagnostic feature of fibrous continuity between the leaflets of the pulmonary valve and the left-sided tricuspid valve. This type of defect extends posteriorly and inferiorly towards the crux of the heart, opening into the inlet of the morphologically left ventricle. An extensive area of fibrous continuity between the leaflets of the pulmonary, mitral, and tricuspid valves forms the postero-superior margin of the ventricular septal defect.

Muscular defects can also be found in any part of the ventricular septum, while doubly committed, or so-called subarterial or "supracristal" defects, can be more common in the oriental populations.

### Obstruction of the Pulmonary Outflow Tract

The anatomical nature of the obstruction to the flow of blood to the lungs can be valvar or subvalvar. The subvalvar stenosis can be muscular or fibrous, related to muscular hypertrophy, presence of a fibrous shelf on the septum, or aneurismal dilation of fibrous tissue derived from the interventricular component of the membranous septum or an atrioventricular valve. When present, such subvalvar obstructions are intimately related to the non-branching atrioventricular bundle. Pulmonary atresia can also be seen in the setting of congenitally corrected transposition, representing the extreme form of pulmonary obstruction.

### Lesions of the Morphologically Tricuspid Valve

**Figure 9:**
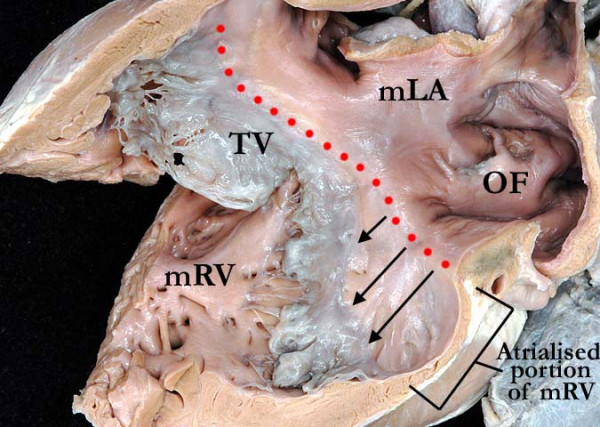
**This illustration demonstrates the apical displacement (lines) of the septal and mural leaflets of the tricuspid valve (TV)**. The morphologically left atrium (mLA) is connected to the morphologically right ventricle (mRV) through an Ebstinoid-like tricuspid valve. Note the flap valve of the oval fossa on the atrial septal surface.

The most common underlying pathology of the morphologically tricuspid valve is the apical displacement of the septal and mural leaflets (Figure 9). This justifies the description of Ebstein's malformation. The septal leaflet is displaced inferiorly towards the cardiac apex [7], but it is rare to find the "sail - like" deformity of the antero-superior leaflet characteristic of the malformation as seen in the setting of concordant atrioventricular connections. The atrialization of the inlet of the morphologically right ventricle is also minimal, and the clinical symptomatology depends more on the extent of regurgitation across the valve.

Close to three-quarters of patients with tricuspid valvar abnormalities also have ventricular septal defects. Another morphological variation is straddling of the tricuspid valve, with its orifice overriding the ventricular septum in the presence of a ventricular septal defect, with variable hypoplasia of the morphologically right ventricle depending on the commitment of the overriding component of the valve to the morphologically left ventricle. The situation becomes double inlet left ventricle with incomplete left-sided right ventricle when more that half of the overriding atrioventricular valve is connected to the morphologically left ventricle. The morphologically mitral valve can also straddle and override, often in combination with double outlet right ventricle [8]^.^

### Other Ventriculo-arterial Connections

Although the ventriculo-arterial connections are of necessity discordant in the setting of congenitally corrected transposition, the discordant atrioventricular connections can be found with variable ventriculo-arterial connections. The possibilities include concordant ventriculo-arterial connections, producing the physiology of transposition, as well as double outlet from either ventricle, usually the right, or single outlet from the heart. Single outlet can exist as a common arterial trunk, a single pulmonary trunk with atresia of the aortic trunk, or a single aortic trunk with pulmonary atresia. The latter is most common [9]. In the presence of pulmonary atresia, there is typically a large ventricular septal defect in subaortic position. The associated defects will impact on the choice of procedure for surgical management, if this is feasible. This will be expanded on in our section devoted to surgical management.

## Morphogenesis

The chambers of the heart develop by ballooning from the primary heart tube, with the atrial appendages ballooning in parallel from the atrial component of the tube, while the apical component of the ventricles balloon in series from the inlet and outlet parts of the ventricular loop. When development occurs in the normal fashion, the primary heart tube turns to the right during the early stages of development, with the atrioventricular canal then connected primarily to the part of the loop which will form the morphologically left ventricle. Expansion of the canal to the right permits the right atrium to connect directly to the developing morphologically right ventricle. Should the primary heart tube turn leftward instead of to the right, the outlet component, from which the morphologically right ventricle develops, will be placed to the left of the developing left ventricle, so that the morphological left atrium is placed in communication with the morphologically right ventricle subsequent to expansion of the atrioventricular canal. It is not known, however, why such abnormal looping should also be associated so frequently with the development of discordant ventriculo-arterial connections.

## Clinical Description

Symptomatology, if present in the newborn, infant, and child, is related to the associated malformations. If there are no associated defects, which is uncommon, the patient will typically be asymptomatic early in life. Cyanosis will be present if there is pulmonary stenosis and a ventricular septal defect, while heart failure will develop earlier in life if there is a hemodynamically significant ventricular septal defect, evidenced in children by easy fatigability, poor weight gain, feeding intolerance, and so on.

The diagnosis can be made, by serendipity, on the basis of a chest radiograph or electrocardiogram performed for another reason. Diagnosis can also be made later in childhood, or as an adult, when patients present with complete heart block or cardiac failure, even though systemic right ventricular failure without any associated cardiac defects is rarely seen. Some patients can live a full life without any problems, the entity being discovered as a chance finding at autopsy.

On physical examination, the most common presenting features are bradycardia related to high-degree atrioventricular block, and auscultation of a single loud second heart sound, which is often palpable to the left sternal border because of the anteriorly positioned aortic valve. A murmur will be present is there is an associated ventricular septal defect, pulmonary stenosis, or tricuspid regurgitation [10].

Complete atrioventricular block occurs in up to one-third of patients. It can be present at birth, or it can develop at a rate of 2% per year. Other conduction disturbances described include sick sinus syndrome, atrial flutter, re-entrant atrioventricular tachycardia due to an accessory pathway along the atrioventricular junctions, and ventricular tachycardia.

## Natural History

The natural history of a pathology as complex as congenitally corrected transposition is defined by the associated malformations, and by the timing and approach to surgical palliative repair.

Approximately one-tenth of infants born with congenitally corrected transposition have complete heart block [11,12]. In patients born with normal cardiac conduction, the risk of developing heart block over time increases by 2% per year until it reaches a prevalence of 10 to 15% by adolescence, and 30% in adulthood [13]. Around two-fifths of adult patients, nonetheless, will have normal cardiac conduction throughout their lives. The etiology of the disordered conduction is related to the abnormal position of the atrioventricular node and the conduction axis. As time passes, the PR interval prolongs, until complete heart block becomes manifest [14].

Several multicentric studies have found an increasing incidence of cardiac failure with age. By the age of 45 years, half of the patients with associated lesions, and one-third of those without significant associated lesions, present with dysfunction of the systemic morphologically right ventricle. In the setting of congenitally corrected transposition without any other associated anomalies, the ventricular function is adequate to maintain a "normal" activity level into adult life [15,16], although the function of the systemic right ventricle tends to deteriorate gradually after the second decade of life [17].

The systemic morphologically right ventricle tends to respond differently to exercise in comparison to a normal morphologically left ventricle. It is known that the organization of the myocytes is different between the right and left ventricles. The left ventricular myocytes are arranged in layers of counter-wound helixes that surround the ventricular cavity, conferring a special twisting motion during systole and early diastole [18], and providing the optimal stress and strain to generate the necessary forces to sustain the demand on a systemic ventricle. The morphologically right ventricle lacks the helical myocytic arrangement, lacking also the twisting or torsion component conferred by the helical arrangement, and thus being unable to sustain the demands of a systemic ventricle. When in systemic position, the morphologically right ventricle is also unable to respond to an increasing work demand, such as exercise, in the fashion of the normal systemic ventricle [19].

Systemic ventricular dysfunction is also caused by volume overload due to atrioventricular valvar regurgitation or abnormal myocardial perfusion, with ischemia of the myocardium during periods of increasing demand underscoring the abnormal myocardial function [20,21].

The presence of associated malformations further affects the development of myocardial dysfunction and heart block. Patients with large ventricular septal defects and hemodynamically significant atrioventricular valvar regurgitation will develop early heart failure [22]. When significant pulmonary stenosis coexists with a ventricular septal defect, cyanosis will develop earlier in life.

The natural history of the left-sided morphologically tricuspid valve is variable. The valve tends to remain competent during the first decade of life, but slowly becomes progressively incompetent during the second to fifth decades of life. If there is an Ebstinoid malformation of the valve, the regurgitation can be seen at birth.

## Diagnosis

### Chest Radiograph

**Figure 10:**
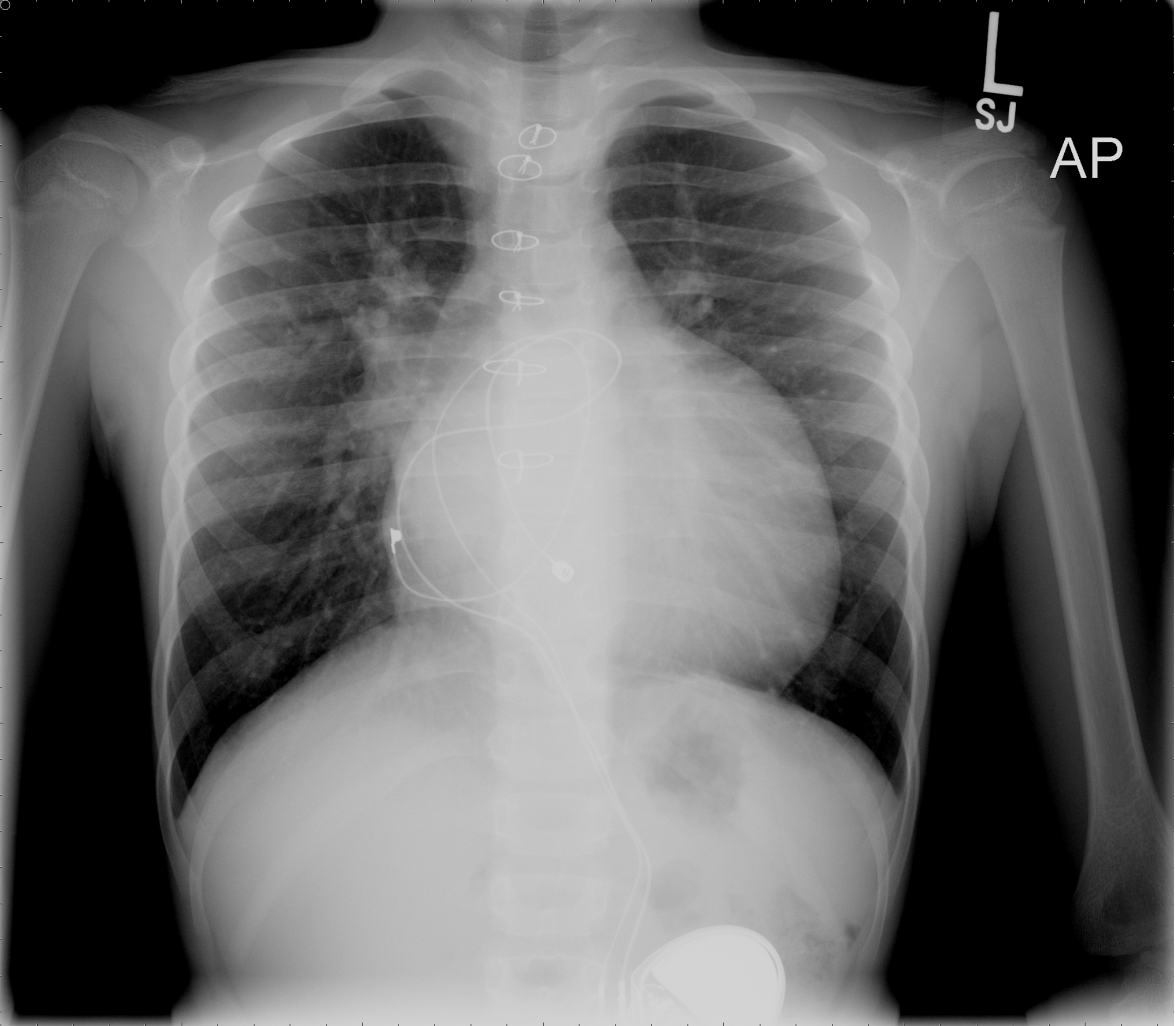
**The upper part of cardiac silhouette as seen in the chest radiograph appears abnormally straight because of the loss of the normal arterial relationships**.

The chest radiograph may show the heart to be in the middle of the chest, or right-sided. The upper part of cardiac silhouette, formed by the aorta and the pulmonary trunk, appears abnormally straight because of the loss of the normal relationships between the arterial trunks (Figure 10). The ascending aorta is typically not visible on the right side, and the convexities from the descending aortic knob and pulmonary trunk are absent on the left side. With usual atrial arrangement, the ventricular border on the left will appear straighter due to the arrangement of the great arteries.

### Electrocardiogram

**Figure 11:**
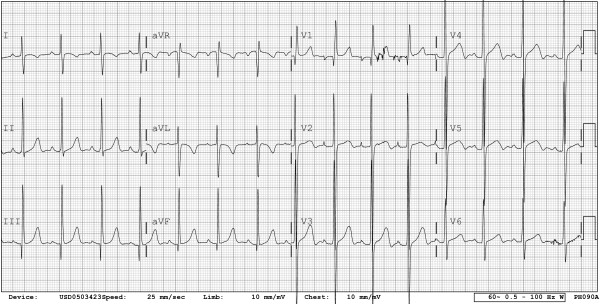
**The electrocardiogram shows a qR pattern in lead V1, with absence of Q waves in leads V5 and V6, demonstrating abnormal ventricular depolarization**.

Due to the mirror-imaged arrangement of the bundle branches in the presence of left handed ventricular topology, the initial activation of the ventricles will be from right to left, represented in the electrocardiogram by Q waves in the right precordial leads, and an absent Q wave in the left precordial leads (Figure 11). This can be erroneously interpreted as an inferior myocardial infarction. Other important findings are related to atrioventricular block or preexcitation.

### Echocardiography

The echocardiogram will confirm the diagnosis, and will determine the presence of any associated malformations, as well as showing the specific relations of the different segments of the heart.

Using a segmental approach to the echocardiogram, the cardiologist will evaluate all the chambers of the heart, their connections, their relationships, and will identify any associated lesions accompanying the abnormal segmental connections.

**Figure 12:**
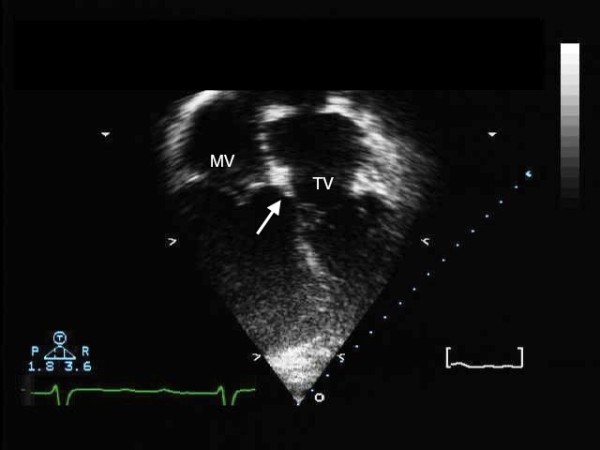
**The four-chamber transthoracic echocardiogram shows reversed off-setting of the leaflets of the mitral and tricuspid valves**.

The subcostal view will be useful in determining the location of the apex of the heart. The apical 4-chamber view (Figure 12), and the short axis cuts, are useful in evaluating the characteristics of the ventricles. Special detail has to be given to the pattern of the apical ventricular trabeculations, since these features determine the morphology of the ventricles.

**Figure 13:**
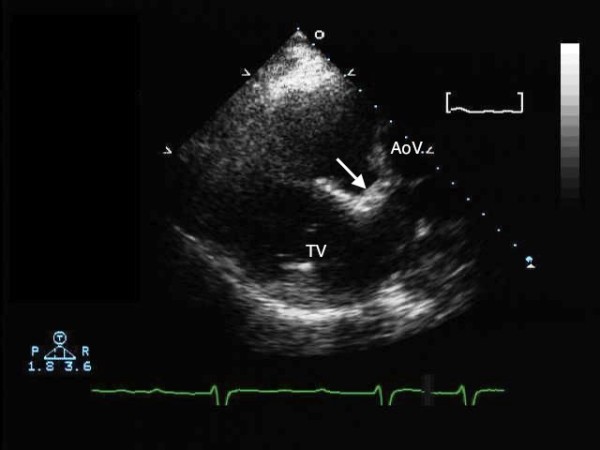
**The parasternal long axis transthoracic echocardiogram shows the subaortic infundibulum (arrow), with lack of fibrous continuity between the left-sided tricuspid (TV) and aortic valves (AoV)**.

If the pattern of ventricular trabeculations remains uncertain, identifying the reverse offsetting of the septal attachments of the leaflets of the tricuspid and mitral valves will confirm the arrangement of the ventricles. In presence of a ventricular septal defect, however, this reversed offsetting can be lost. Fibrous continuity will be seen between the leaflets of the mitral and the pulmonary valves in the long axis and 4-chamber views, and the presence of the subaortic infundibulum helps to identify the morphologically right ventricle (Figure 13).

The relationships of the arterial trunks and valves can be evaluated using the high parasternal view. The aorta in most instances, but not always, will be anterior and to the left of the pulmonary trunk. The coronary arterial anatomy should be delineated using the parasternal short axis view. The usual arrangement is for the anterior descending artery to arise from the right-sided coronary artery.

## Prenatal Diagnosis

Suspicious pregnancies are usually referred to the fetal echosonographer because of a positive family history of congenital heart disease, or when other congenital heart defects have been found during fetal screening. The diagnosis of congenitally corrected transposition can readily be made during fetal life by an experienced sonographer. A recent study demonstrated that several features should alert the sonographer to the possibility of congenitally corrected transposition [23]. The reversed off-setting of the atrioventricular valves was seen in three-quarters of fetuses, while the moderator band was identified in the left-sided or posterior ventricle in almost nine-tenths. The diagnosis becomes more difficult when there is isolated corrected transposition, since there are no other defects in the heart that will prompt the sonographer to carry out a detailed sequential evaluation.

### Cardiac Catheterization

The indications for cardiac catheterization are limited nowadays, since echocardiography and magnetic resonance imaging better delineate the anatomy, ventricular function, and the severity of atrioventricular valvar regurgitation. The need for assessment of the pulmonary vascular resistance, or for angiographic evaluation of the coronary arteries or the aortic arch, complex pulmonary atresia, or pulmonary venous abnormalities are potential indications for an invasive study.

If deemed necessary, cardiac catheterization will confirm the degree of atrioventricular valvar regurgitation, and delineate other associated anatomical malformations of the heart. It also permits the hemodynamic evaluation of the systemic ventricular function, as well as measuring and calculating pulmonary and systemic flows. Evaluation of the coronary arteries should also be performed in older patients prior to any surgical repair so as to delineate their anatomy and distribution, and to evaluate for coronary arterial disease and osteal stenosis [24].

The approach to the catheterization can be technically difficult due to the morphological relationships of the left ventricle and the pulmonary trunk. The initial approach is from the inferior caval vein, the right atrium and through the mitral valve into the morphologically left ventricle. The plane of the mitral valve is oblique rather than vertical, making the pass of the catheter from the right atrium to the pulmonary trunk more difficult than in patients with concordant atrioventricular connections. In addition, the atrioventricular conduction axis is more vulnerable due to its anteriosuperior location, presenting the possibility for traumatic injury with the catheter pass, and thus creating transient atrioventricular block.

If the left-sided chambers cannot be accessed through a patent oval foramen or a ventricular septal defect, a retrograde approach though the aorta into the morphologically right ventricle may be necessary. The left atrium can also be accessed retrogradely by passing the catheter across the tricuspid valve, or alternatively using a transseptal technique.

Full hemodynamic evaluation and anatomic delineation should be the goal of the study. In isolated congenitally corrected transposition, the hemodynamics can be normal. As discussed, however, associated defects are almost always present, leading to predictable hemodynamic alterations.

The morphologically right ventricle is exposed over time to the systemic vascular resistance, potentially causing systemic ventricular dysfunction.

The cardiac output should be measured by thermodilution or by the Fick method, allowing calculation of the systemic and pulmonary vascular resistances.

### Magnetic resonance imaging

**Figure 14:**
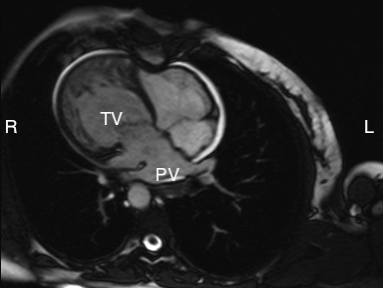
**The magnetic resonance image shows a right-sided heart with the apex pointing to the right**. The pulmonary veins are seen entering the left atrium (PV), with the tricuspid valve [TV] guarding the junction with the coarsely trabeculated systemic morphologically right ventricle.

Magnetic resonance imaging can evaluate in great detail the segmental connections of the heart, permit quantification of systemic and pulmonary blood flows, and assess the severity of atrioventricular valvar regurgitation and ventricular function. The technique requires familiarity with congenitally malformed hearts so that imaging planes are optimized during the procedure. (Figure 14)

## Differential diagnosis

The differential diagnosis is centred on assessing whether the patient is presenting with isolated malformations or as part of a spectrum. The anatomical characteristics of the different segments of the heart and the relations between them will provide the necessary clues to make the diagnosis. In the older patient presenting with unexplained heart failure, an evaluation of ventricular morphology will be crucial.

## Management

### Medical Management

This involves management of the failing systemic right ventricle [24], with timing depending on the severity of the associated malformations [25]. It involves treatment with diuretics, inhibitors of angiotensin converting enzyme, and digoxin. If there are conduction abnormalities, the patient might require implantation of a pacemaker, for example in the setting of advanced second or third degree AV block, or in the presence of symptoms or ventricular dysfunction. If bacterial endocarditis develops, treatment should follow the current guidelines; treatment is recommended if intracardiac lesions remain unrepaired, if prosthetic material has been inserted and the attack occurs within the first 6 months after the procedure, if there is any residual defect with prosthetic material, or if a prosthetic cardiac valve has been used as part of the repair [26].

### Surgical Management

The diagnosis of congenitally corrected transposition is not in itself an indication for surgical correction. If needed, however, surgical treatment in the past was initially dictated by a physiological approach, with repair of the associated lesions. Nowadays, there is an increasing trend to achieve anatomical restoration of the circulation, making the morphologically left ventricle the systemic pumping chamber. If the decision is made to achieve anatomic correction, the timing of surgery is always complex, especially in asymptomatic patients. The decision should be based on the potential of the systemic right ventricle to fail, the hemodynamic impact of the associated lesions, and the risk and benefits of operating based on the age and size of the patient. If the systemic ventricle is left exposed to systemic vascular resistance, the increased workload will cause progressive hypertrophy and cavitary enlargement, with deterioration in function such that ultimately the chances of recovery might be small.

When a ventricular septal defect is present, the indications for operation will be dictated by its hemodynamic impact, as well as the specific morphology of the defect itself. In the presence of pulmonary stenosis the indication for surgery is dictated by the severity of the obstruction, and the degree of cyanosis, hypoxemia and polycythemia caused by the right-to-left shunt and the decreased flow of blood to the lungs.

Other indications for surgery include the severity of tricuspid valvar regurgitation and the presence of complete heart block. The anatomical details encountered will ultimately determine the surgical repair. Replacement of the morphologically tricuspid valve is usually recommended should the valve regurgitation become severe, as the anatomical configuration does not lend itself to repair.

When pursuing the physiological approach, the goal of the operation is to repair the cardiac defects by closing any ventricular septal defect, repairing the tricuspid valve, and alleviating any obstruction to pulmonary flow, while leaving the morphologically right ventricle as the systemic ventricle. If heart block occurs during the surgical procedure, it is necessary to insert a pacemaker. The physiologic approach is straightforward but has the drawback that, over time, the systemic right ventricle will likely fail. Because of this poor prognosis over the long term, there is an increasing trend towards achieving anatomical correction. The goal of the anatomical correction is the rerouting of the pulmonary venous return to the morphologically left ventricle and aorta, and the systemic venous return to the morphologically right ventricle and pulmonary arteries, thus achieving a normal anatomic pattern of circulation. This can be achieved by baffling the systemic venous return to the morphologically right ventricle and the pulmonary venous return to the morphologically left ventricle at atrial level, combining this procedure with either an arterial switch procedure or an interventricular baffle.

The feasibility of such double switch procedures will depend on the associated cardiac malformations. In patients with pulmonary stenosis and a ventricular septal defect, it is possible to place an interventricular baffle to redirect the flow to the aorta, placing a valved conduit between the morphologically right ventricle and the pulmonary arteries. When no pulmonary stenosis is present, an arterial switch operation is needed to correct the discordant ventriculo-arterial connections. Relative contraindications to the double switch include anomalous coronary arterial anatomy, hypoplasia of the morphologically left ventricle, extensive straddling of the tricuspid valve, and anomalies of the mitral valve.

The complications of this procedure are related to the atrial switch, and include sinus nodal dysfunction, supraventricular arrhythmias, and problems with the atrial baffle. If the ventricular switch is used, aortic obstruction, aortic regurgitation, or conduit obstruction and regurgitation can all occur. If an arterial switch is done as part of the double switch, coronary arterial obstruction or stenosis, aortic valvar regurgitation, and pulmonary arterial stenosis can occur. If a homograft conduit is used to restore the circulation to the pulmonary arteries, deterioration of the homograft valve will require replacement. Those performing the double switch procedure in large centers have produced good intermediate results, with early mortality currently reported at 5%, and long-term survival at 10 years of up to 95% [27].

If it is not possible to achieve a biventricular repair, the surgical approach should be staged to result in creation of the Fontan circulation at the appropriate age based on the size and weight of the patient.

Placement of a pacemaker is often required during surgery, since up to one-quarter of patients have been reported as developing complete heart block [28], although knowledge of the disposition of the conduction tissue should allow these complications to be avoided. Replacement of the systemic atrioventricular valve is needed with some frequency in adults should there be significant atrioventricular valvar regurgitation.

### Follow up

Follow up is ideally performed in a center with a programme for adult congenital heart disease run by pediatric cardiologists, or by an adult congenital cardiologist who has had special training and experience caring for patients with congenital heart disease. It should be done in a periodic and regular fashion. The evaluation consists of an echocardiogram and/or magnetic resonance imaging to assess the anatomical relationships changed by the surgical repair, along with assessment of the systolic ventricular function and the degree of atrioventricular valvar regurgitation. Exercise tolerance can be studied with a treadmill exercise test. Any abnormality in rhythm warrants immediate evaluation, ideally by an electrophysiologist.

## Prognosis

There have been some reports of patients with congenitally corrected transposition living into late adulthood without heart failure in the absence of associated lesions [29], but this is not the norm. More usually, the patient will develop progressive insufficiency of the tricuspid valve, with volume overload of the morphologically right ventricle leading to progressive heart failure, once the ventricle is unable to meet the demands of the systemic circulation. Surgical intervention is then necessary to decrease the deleterious effects of the associated malformations, and delay the progress of cardiac failure.

## Abbreviations

(Ao): Terms used: aorta; (CS): coronary sinus; (OF): oval fossa, (mLA): morphologically left atrium; (mRA): morphologically right atrium; (MV): mitral valve; (mLV): morphologically left ventricle; (mRV): morphologically right ventricle; (VSD): ventricular septal defect; (PT): pulmonary trunk; (PV): pulmonary veins, (SCV): superior caval vein; (ICV): inferior caval vein; (TV): tricuspid valve.

## Competing interests

The authors declare that they have no competing interests.

## Authors' contributions

GW researched the natural history, clinical symptomatology, diagnosis, differential diagnosis, management, follow up and prognosis. DA contributed with the anatomy, morphogenesis and the revision and editing of the manuscript. DDS contributed with the preparation and photography of the cardiac specimens depicted in the manuscript. All authors read and approved the final manuscript.
